# Cancer‐associated fibroblasts: activin A adds another string to their bow

**DOI:** 10.15252/emmm.202012102

**Published:** 2020-03-26

**Authors:** Remi Samain, Victoria Sanz‐Moreno

**Affiliations:** ^1^ Barts Cancer Institute John Vane Science Centre Queen Mary University of London London UK

**Keywords:** Cancer, Skin

## Abstract

Non‐melanoma skin cancer (NMSC) is characterized by a strong desmoplastic reaction, largely responsible for cancer aggressiveness. Within the tumour microenvironment, cancer‐associated fibroblasts (CAFs) play a key role in tumour progression, secretion of extracellular matrix proteins and recruitment of immunosuppressive cells. However, pathways involved in acquisition of CAF phenotype remain unclear. In this issue of *EMBO Molecular Medicine*, Cangkrama *et al* describe a new mechanism of fibroblast activation in squamous cell carcinoma. Cancer cell‐secreted activin A induces a tumour‐promoting phenotype in the fibroblast compartment, with distinct properties compared to TGF‐β‐activated fibroblasts. Activin A reprograms fibroblasts through transcriptional regulation of mDia2 and reduction of nuclear p53, which favours CAF marker expression, and increases tumour growth and migration. Inhibition of this pathway shows promising results in different models and could offer a new therapeutic strategy in NMSC.

## Graphical abstract

V. Sanz‐Moreno and R. Samain discuss a new mechanism of fibroblast activation mediated by squamous cell carcinoma‐secreted Activin A, and whose inhibition shows promising results in non‐melanoma skin cancer (Cangkrama *et al*, this issue of *EMBO Mol Med*).

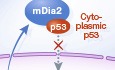

The incidence of non‐melanoma skin cancer (NMSC)—a highly frequent malignancy—has dramatically increased in the last decades (Lomas *et al*, 2012). Thus, understanding the biology of these tumours is fundamental to improve patient treatment. As in many other solid tumours, NMSC is characterized by an active tumour microenvironment (TME) that controls cancer aggressiveness and progression (Bordignon *et al*, 2019). Increasing evidence shows that cross‐talk between cancer and stromal cells stimulates tumorigenesis, providing opportunities for the development of new therapeutic strategies.

The TME is composed of an acellular part, characterized by a dense network of extracellular matrix (ECM) proteins comprising mainly collagens, and of several cellular components (Vennin *et al*, 2018). Among them are cancer‐associated fibroblasts (CAFs), master secretors of ECM proteins, and involved in the recruitment of immunosuppressive cell populations: tumour‐associated macrophages or regulatory T lymphocytes (Sahai *et al*, 2020). Cancer cells also activate these TME components, which in turn accelerate tumour growth and resistance to treatments. Therefore, a better characterization of how cancer cells communicate with the TME is crucial for next‐generation therapies.

The means used by cancer cells to activate differentiation of normal fibroblasts into CAFs are critical, given the key role of these cells in cancer progression. Many potential molecular players contributing to CAF activation have been described, notably TGF‐β itself, inflammatory modulators (interleukins), DNA damage or stimulation of tyrosine kinase receptors by growth factors (Sahai *et al*, 2020). In this issue of *EMBO Molecular Medicine*, Cangkrama *et al* (2020) elegantly describe how activin A, a transforming growth factor β (TGF‐β) family member, induces tumour‐promoting fibroblast phenotypes. The pro‐tumourigenic features of CAFs have been well described and range from CAF secretion of soluble factors and ECM proteins (Duluc *et al*, 2015) to mechanical interactions with cancer cells contributing to tumour invasion (Gaggioli *et al*, 2007; Sanz‐Moreno *et al*, 2011). Nevertheless, the pathways involved in establishing a CAF phenotype are unclear. Moreover, CAF depletion can have opposite effects and favour tumour growth (Rhim *et al*, 2014), suggesting that reprograming of CAFs into a less activated state could offer higher therapeutic benefits. Cangkrama *et al* (2020) recent findings are in line with these observations.

This comprehensive study combines organotypic assays, cell and molecular biology, *in vivo* models and transcriptomics. Using primary human dermal fibroblasts, the authors observed differential activation of fibroblasts by activin A compared to TGF‐β. This activation was associated with increased filopodial length and migrating abilities, suggesting the induction of a different CAF subtype (Elyada *et al*, 2019). These activated fibroblasts were able to secrete large amounts of ECM proteins and to favour both migration and clonogenicity of squamous cell carcinoma (SCC) cells. Fibroblasts from mice developing skin papillomas crossed with mice overexpressing *INHBA* (activin A encoding gene) in keratinocytes were characterized using transcriptomics, and an enrichment for genes associated with activated fibroblasts was observed in mice overexpressing activin A. Among these genes, the authors identified the cytoskeletal regulator *mDia2* as strongly activated by activin A compared to TGF‐β. Mechanistically, activin A promoted the binding of SMAD2/3 to a “SMAD Binding Element” identified in the first intron of the *mDia2* gene, which was not the case with TGF‐β. mDia2 protein was strongly expressed in the stroma of NMSC and its expression negatively correlated with survival of patients in several cancers. Interestingly, in such fibroblasts, mDia2 was able to physically interact with p53, leading to p53 nuclear inhibition. Moreover, mDia2 silencing in fibroblasts led to a decrease of CAF marker gene expression, and to blocking of fibroblast tumorigenic properties *in vivo*. Finally, the authors confirmed that the activin A/mDia2 axis could be targeted by both follistatin (an activin A antagonist) and SMIFH2 (an mDia2 inhibitor), which led to decreased proliferation of cancer cells (Fig. 1).

**Figure 1:**
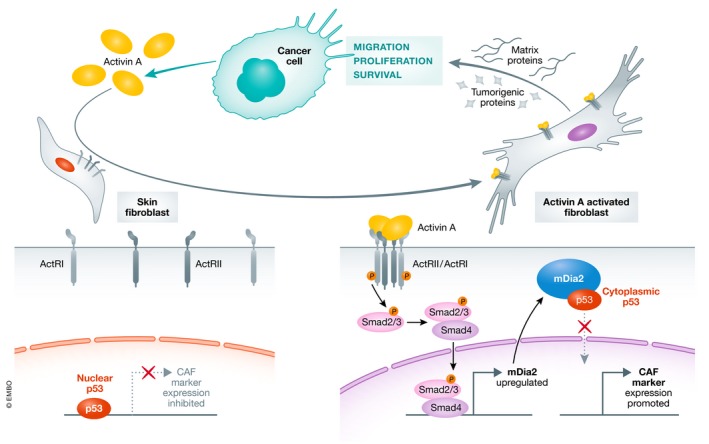
A vicious circle between cancer cells and fibroblasts Cancer cells secrete activin A, which activates skin fibroblasts through SMAD2/3 pathway and mDia2 upregulation. mDia2 interacts with p53 to reduce nuclear p53 and therefore promotes CAF marker expression. In turn, activin A‐activated fibroblasts support cancer cell survival, proliferation and migration.

Taken together, the study conducted by Cangkrama *et al* sheds light on a new signalling pathway responsible of CAF activation in skin cancer, which could potentially be targeted at different levels. Such strategy could benefit other cancer types associated with CAF infiltration, including colorectal or breast cancer. Tumour microenvironment is a new recognized hallmark of cancer, and its targeting has opened the door to new therapies. Time will tell whether such therapeutic approaches live up to our expectations.
